# Sexual awareness, high-risk sexual behaviors and HIV testing: a cross-sectional survey among university students in Xuzhou, Jiangsu

**DOI:** 10.3389/fpubh.2025.1578062

**Published:** 2025-05-21

**Authors:** Hualing Li, Qi Wu, Qinghan Li, Enze Gao, Dehui Yin

**Affiliations:** ^1^School of Public Health, Xuzhou Medical University, Xuzhou, Jiangsu, China; ^2^Discipline Inspection Commission, Xuzhou Medical University, Xuzhou, Jiangsu, China

**Keywords:** university students, gender psychology, sexual behavior, high-risk sexual behavior, HIV testing

## Abstract

**Background:**

As societal dynamics evolve, a noticeable trend has emerged indicating that Chinese adolescents are engaging in sexual activities at increasingly younger ages. This demographic is therefore identified as a crucial group for initiatives aimed at the prevention and control of HIV. The primary objective of this study is to investigate the psychological and behavioral characteristics of gender (male and female) college students in Xuzhou. It will analyze the key factors that influence their sexual behaviors, particularly those associated with high-risk sexual practices, and explore the determinants that affect their willingness to participate in HIV testing. This research aims to establish a scientific foundation for enhancing college students’ understanding of HIV prevention and control strategies, ultimately contributing to a reduction in the risk of infection.

**Methods:**

This research employed a cross-sectional survey design to distribute an anonymous questionnaire to a sample of 4,193 college students from four universities in Xuzhou. The questionnaire included items related to demographic characteristics, gender-specific psychological perceptions, sexual behavior patterns, and HIV testing status. Univariate analysis was conducted using chi-square tests, while logistic regression was utilized to examine the interactive effects of multiple variables.

**Results:**

In the studied population, males comprised 44.0%, while females accounted for 56.0%, with medical students representing 55.8% of the sample. The survey revealed that 9.3% of students reported having sexual experience, and 3.27% engaged in high-risk sexual behaviors, with a notably higher prevalence among males compared to females. Multivariate analysis identified several key factors influencing high-risk sexual behavior, including gender, academic grade level, monthly expenditure, sexual orientation, and dating experience. Females were found to have a lower likelihood of engaging in high-risk sexual behavior compared to their male counterparts (OR = 0.72, 95% CI = 0.122–1.623). In contrast, the risk was significantly higher for homosexual (OR = 3.12, 95% CI = 1.607–6.052) and bisexual (OR = 2.64, 95% CI = 1.289–5.423) students. Furthermore, upperclassmen and those with multiple dating experiences exhibited correspondingly increased risks. The prevalence of HIV testing within the population was 4.6%, with influencing factors including gender, whether the student is an only child, ethnicity, sexual orientation, dating experience, and previous sexual behavior. Notably, the willingness to undergo testing was lower among females, ethnic minorities, and individuals identifying as bisexual.

**Conclusion:**

This research examines the current conditions and characteristics of college students in Xuzhou concerning sexual psychology, sexual behavior, and HIV testing. It is advisable for universities to establish focused sexual health education and HIV prevention programs that are specifically designed to address the unique attributes of various student demographics, with particular attention to high-risk groups. Additionally, institutions should ensure the availability of accessible testing services, create a robust psychological support framework, reduce the risk of HIV transmission, and promote a healthy and safe campus atmosphere.

## 1 Introduction

Acquired Immunodeficiency Syndrome (AIDS) is a profoundly detrimental and lethal disease that compromises the immune system, significantly affecting global public health and imposing substantial economic challenges, particularly in low- and middle-income countries (1). The most recent report published by the Joint United Nations Programme on HIV/AIDS (UNAIDS) in 2023 reveals that in 2022, approximately 39 million individuals were living with Human Immunodeficiency Virus (HIV) worldwide, with 1.3 million new infections reported, 23% of which were concentrated in the Asia-Pacific region (2). Data from the Chinese Center for Disease Control and Prevention (CDC) indicate that from 2010 to 2019, the number of newly reported HIV infections among young students in China totaled 23,307 (3), with annual new cases remaining at approximately 3,000 per year and a growth rate ranging from 30 to 50% (4). Notably, Jiangsu Province ranked second in the nation for cumulative reported cases during this period. In order to effectively mitigate the transmission of HIV among young students, the National Health Commission and other relevant departments have articulated in the “Implementation Plan for Containing the Spread of AIDS (2019–2022)” (5), the necessity of enhancing multi-faceted HIV interventions within universities. As a prominent city in northern Jiangsu Province, Xuzhou is home to numerous universities and boasts a well-developed transportation network, which facilitates the rapid spread of HIV (6). Consequently, conducting a survey on sexual psychology and behavior, high-risk sexual practices, and HIV testing among college students in this region is of considerable strategic significance.

With advancements in science, technology, and information networks, the age at which Chinese adolescents reach sexual debut is progressively decreasing, accompanied by a discernible trend of younger individuals, particularly adolescents, engaging in sexual activities (7). In this context, the population of college students, who are in a phase of increased sexual exploration and activity, is also experiencing an annual rise in the prevalence of sexual behavior (8).

Young students exhibit relatively permissive attitudes toward sexual matters, which may predispose them to engage in high-risk sexual behaviors. This propensity significantly increases the risk of contracting sexually transmitted infections (STIs), including HIV, syphilis, and gonorrhea (9), resulting in physical suffering and placing a significant economic burden on individuals. Furthermore, such behaviors may precipitate long-term psychological consequences (10), including depression, anxiety, and identity crises. A survey indicated that 37.3% of male college students in China reported having multiple sexual partners within the preceding 6 months (11), while reported engaging in unsafe sexual practices, such as unprotected intercourse (12). These findings suggest that young students in China lack sufficient awareness of the need to prevent high-risk sexual behaviors and have a limited understanding of the potential risks associated with infection, thereby creating an environment conducive to the transmission of HIV (13). Currently, HIV counseling and testing (HCT) is regarded as a critical strategy for combating the spread of HIV. HCT serves not only as a fundamental component of primary prevention for HIV, effectively reducing virus transmission, but it also facilitates the early detection and diagnosis of infections. This early intervention is essential for slowing disease progression and enabling timely treatment and care (14). Nevertheless, numerous studies have consistently demonstrated that the use of HIV testing services among Chinese college students remains low, and the distribution of HCT resources within this demographic is uneven. This disparity poses significant challenges to HIV prevention and control efforts among Chinese college students (15, 16).

This study aims to achieve a multi-faceted understanding of the sexual psychology and behavioral patterns of college students in Xuzhou. It seeks to analyze the key factors influencing college students’ participation in sexual activities, particularly high-risk sexual behavior. The objective is to provide a scientific foundation for developing targeted prevention and intervention strategies to reduce the prevalence of high-risk sexual behavior. Furthermore, this research investigates the factors affecting college students’ uptake of HIV testing services, aiming to improve understanding of HIV prevention awareness and related behaviors within the college student demographic. Ultimately, this study aims to provide valuable insights for implementing effective measures to reduce the risk of HIV infection.

## 2 Materials and methods

### 2.1 Data sources

This cross-sectional study was conducted from September to November 2023 in four universities in Xuzhou, Jiangsu Province, China, using cluster random sampling. The study protocol was approved by the Ethics Committee of Xuzhou Medical University, and all participants provided electronic informed consent forms. Data collection was conducted using the WJX platform,^1^ adhering strictly to principles of voluntariness, confidentiality, and anonymity throughout the process.

On-campus students completed the electronic questionnaire by scanning a QR code on mobile devices under the supervision of their teachers, while internship students accessed the survey through dedicated links. The informed consent process included: (1) Presentation of the research objectives, privacy protections, and a statement on voluntary participation on the questionnaire’s introductory page; (2) Mandatory checkbox confirmation (“I have read and agree to participate”) before survey access; (3) Option to withdraw at any time without data retention. The study complied with the ethical guidelines for human subject research outlined in the Declaration of Helsinki.

**Figure 1:**
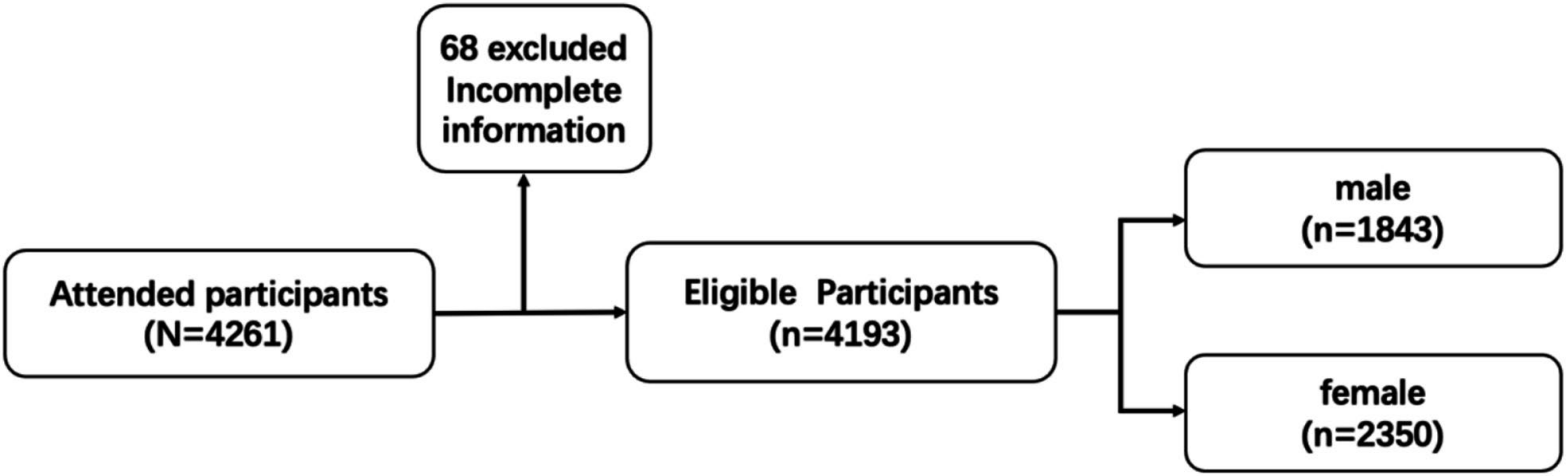
Flowchart of study participants.

The initial study included 4,261 participants, of whom 68 were excluded from the analysis. Among those excluded, 63.2% were removed due to incomplete or invalid information, while 36.8% were excluded because the time taken to complete the survey was less than 30 s. As a result, the final study comprised 4,193 participants, yielding a valid questionnaire response rate of 98.4% (Figure 1).

### 2.2 Survey design

A self-developed questionnaire was used to conduct a self-administered anonymous survey focusing on participants’ gender psychology and sexual behavior. The questionnaire consists of three sections.

The initial section collects essential demographic information from participants, including variables such as gender, academic grade, field of study, whether they are an only child, ethnicity, type of residence (urban or rural), and monthly living expenses.

The second section gathers data on participants’ gender psychology and behavioral status, employing a scale adapted from the “Potential Risk Assessment Scale for College Students’ HIV-Related Behaviors” (17), developed by the Chinese Center for Disease Control and Prevention. This section includes three questions related to gender psychology and three questions addressing sexual behavior (18).

The third section collects data related to sexual behavior, including the occurrence of sexual activity, the frequency of condom use, the identities of partners involved in sexual encounters, the administration of HIV testing, and the results of those tests.

High-risk sexual behavior refers to actions that significantly increase the risk of HIV infection. Specifically, it involves unprotected sexual activities with individuals from high-risk groups (19). In this questionnaire, high-risk groups include those who selected “commercial sex partners,” “casual sex partners,” “homosexual partners” or “multiple sexual partners” in the question regarding the type of sexual partner (20). Unprotected sexual behavior is defined as selecting “part-time use “or “not using “in the question about condom usage frequency (21).

### 2.3 Statistical methods

Frequencies and percentages were employed to characterize categorical variables, while the chi-squared (χ^2^) test was utilized for comparisons among two or more groups. Binary logistic regression analysis was used for multifactorial assessments. The factors included in the binary logistic regression analysis were those that were statistically significant in the chi-squared test, and the results were presented as odds ratios (OR) with 95% confidence intervals (CI). All statistical tests were two-tailed, with a significance level set at *α* = 0.05. The analyses were executed using SPSS version 26.0.

## 3 Results

### 3.1 Fundamental demographic characteristics of college students

Among the participants, 1,843 (44.0%) identified as male and 2,350 (56.0%) identified as female. Additionally, 2,338 (55.8%) were medical students, while 1,855 (44.2%) were non-medical students. Regarding grade distribution, freshmen comprised 44.5%, sophomores 24.6%, juniors 21.0%, and seniors and above accounted for 9.9%. The largest proportions were observed among non-only children (2,288, 54.6%), Han Chinese individuals (4,062, 96.9%), the urban population (2,611, 62.3%), and heterosexual individuals (3,432, 81.9%) (22).

### 3.2 Psychological and behavioral cognition of college students across genders

A particular subgroup of the total 4,193 students exhibited emotional attraction toward individuals of the same sex (Table 1). Among the male participants, 2.0% (37 out of 1,843) reported experiencing romantic feelings for those of the same sex. Interestingly, the same percentage was seen among the female participants, with 2.0% (47 out of 2,350) reporting similar feelings. Additionally, 2.2% (41 out of 1,843) of the male students and 2.1% (50 out of 2,350) of the female students indicated a desire for a same-sex romantic partner. Furthermore, 2.4% (45 out of 1,843) of the male students and 3.1% (73 out of 2,350) of the female students expressed a wish to have a lifelong relationship with someone of the same sex. Notably, the proportion of females who were open to partners of both genders across these three aspects was higher than that of males. Moreover, a larger percentage of females reported an inability to accept partners of any gender compared to their male counterparts.

**Table 1:** Psychological cognition of college students by gender [n (%)].

Questions		Male (*n* = 1,843)	Female (*n* = 2,350)
The person who used to make you have heartfelt feelings	Only male	37(2.0)	1,114(47.4)
	Only female	1,470(79.7)	47(2.0)
	Both male and female	154(8.3)	682(29.0)
	None	182(9.9)	507(21.6)
The person you want to be your romantic partner	Only male	41(2.2)	1,258(53.5)
	Only female	1,373(74.4)	50(2.1)
	Both male and female	72(3.9)	208(8.9)
	None	357(19.3)	834(35.5)
The person you want to be with for the rest of your life	Only male	45(2.4)	1,121(47.7)
	Only female	1,521(82.4)	73(3.1)
	Both male and female	104(5.6)	441(18.8)
	None	173(7.4)	715(30.4)

A specific subset of the 4,193 students exhibited a behavioral preference for individuals of the same sex (Table 2). Among the male participants, 1.8% (34/1,843) reported having been or currently being in love with someone of the same sex, while 1.6% (37/2,350) of female participants indicated the same. Additionally, 2.3% (43/1,843) of males and 1.5% (35/2,350) of females reported experiencing pleasure exclusively from physical contact with individuals of the same sex. Furthermore, 2.2% (41/1,843) of males and 1.3% (30/2,350) of females acknowledged having engaged in masturbation while fantasizing about the same sex. Notably, the proportion of females who are open to both male and female partners, or who do not accept any gender, is greater than that of males. For instance, the percentage of females (78.2%, 1,837/2,350) who do not fantasize about any gender during masturbation is more than double that of males (33.9%, 625/1,843).

**Table 2:** Behavioral cognition of college students by gender [n(%)].

Questions		Male (*n* = 1,843)	Female (*n* = 2,350)
The person you have been or are currently in love with	Only male	34(1.8)	999(42.5)
	Only female	1,199(65.0)	37(1.6)
	Both male and female	55(3.0)	154(6.6)
	None	555(30.1)	1,160(49.9)
The person who have given you pleasure by touching your body	Only male	43(2.3)	445(18.9)
	Only female	1,035(56.1)	35(1.5)
	Both male and female	58(3.1)	117(5.0)
	None	707(38.3)	1753(74.6)
The person you have fantasied about while masturbating	Only male	41(2.2)	342(14.6)
	Only female	1,125(61.0)	30(1.3)
	Both male and female	52(2.8)	101(4.3)
	None	625(33.9)	1837(78.2)

### 3.3 Analysis of factors influencing sexual behavior and high-risk sexual behavior among college students

In the surveyed student population, the prevalence of those who reported having engaged in sexual behavior stood at 9.3% (388 out of 4,193 individuals), while the prevalence of high-risk sexual behavior was 3.27% (137 out of 4,193) (Table 3). Specifically, the proportion of male students involved in sexual behavior was 11.9% (220 out of 1,843), as opposed to 7.1% (168 out of 2,350) for female students. Moreover, the prevalence of high-risk sexual behavior among male students was 4.2% (77 out of 220), while for female students, it was 2.6% (60 out of 168). These proportions were significantly higher among males than among females (*p* < 0.001).

**Table 3:** Univariate analysis of factors influencing sexual behavior and high-risk sexual behavior among students.

Variables	N(%)	Sexual behavior			High-risk sexual behavior		
		N(%)	χ^2^	*P*	N(%)	χ^2^	*P*
Gender			28.201	<0.001		8.628	0.003
Male	1843(44.0)	220(11.9)			77(4.2)		
Female	2,350(56.0)	168(7.1)			60(2.6)		
Grade			144.806	<0.001		41.115	<0.001
Freshman	1866(44.5)	94(5.0)			43(2.3)		
Sophomore	1,033(24.6)	95(9.2)			32(3.1)		
Junior	881(21.0)	102(11.6)			27(3.1)		
Senior and more	413(9.9)	97(23.5)			35(8.5)		
Major			2.051	0.152		2.154	0.142
Medical	2,338(55.8)	203(8.7)			68(2.9)		
Non-medical	1855(44.2)	185(10.0)			69(3.7)		
Only child or not			3.597	0.058		2.334	0.127
Yes	1905(45.4)	194(10.2)			71(3.7)		
No	2,288(54.6)	194(8.5)			66(2.9)		
Nationality			44.915	0.001		61.608	<0.001
Han	4,062(96.9)	354(8.7)			117(2.9)		
Minority	131(3.1)	34(26.0)			20(15.3)		
Native place			0.139	0.709		0.171	0.679
Urban	2,611(62.3)	245(9.4)			83(3.2)		
Rural	1,582(37.7)	143(9.0)			54(3.4)		
Monthly expenses			162.131	<0.001		203.182	<0.001
≤ 1,000 CNY	194(4.6)	37(19.1)			22(11.3)		
1,000–2,000 CNY	2,949(70.3)	203(6.9)			57(1.9)		
2,000–3,000 CNY	902(21.5)	96(10.6)			27(3.0)		
≥3,000 CNY	148(3.5)	52(35.1)			31(20.9)		
Sexual orientation			73.862	<0.001		125.227	<0.001
Heterosexual	3,407(82.9)	289(8.5)			75(2.2)		
Homosexual	74(1.8)	24(32.4)			15(17.6)		
Bisexual	220(5.4)	48(21.8)			29(11.7)		
Uncertain	408(9.9)	27(6.6)			18(4.2)		
Number of love affairs			877.242	<0.001		906.932	<0.001
None	3,108(74.1)	78(2.5)			30(1.0)		
1–2 times	954(22.8)	230(24.1)			48(5.0)		
3–5 times	51(1.2)	21(41.2)			12(23.5)		
More than 5 times	80(1.9)	59(73.8)			47(58.8)		
Sex education			0.191	0.662		8.404	0.106
Yes	3,001(71.6)	274(9.1)			83(2.8)		
No	1,192(28.4)	114(9.6)			54(4.5)		

The univariate analysis of sexual behavior revealed that, apart from gender (χ^2^ = 28.201, *p* < 0.001), several other variables were statistically significant. These variables included nationality (χ^2^ = 44.915, *p* = 0.001), grade level (χ^2^ = 144.806, *p* < 0.001), monthly expenses (χ^2^ = 162.131, *p* < 0.001), sexual orientation (χ^2^ = 73.862, *p* < 0.001), and the number of romantic relationships (χ^2^ = 877.242, *p* < 0.001).

The univariate analysis of high-risk sexual behavior indicated that several variables are statistically significant (Table 4): gender (χ^2^ = 8.628, *p* = 0.003), grade (χ^2^ = 41.115, *p* < 0.001), nationality (χ^2^ = 61.608, *p* < 0.001), monthly living expenses (χ^2^ = 203.182, *p* < 0.001), sexual orientation (χ^2^ = 125.227, *p* < 0.001), and the number of romantic relationships (χ^2^ = 906.932, *p* < 0.001). Furthermore, the multivariate regression analysis revealed that gender, grade, monthly living expenses, sexual orientation, and the number of romantic relationships significantly influence college students’ engagement in high-risk sexual behavior. Specifically, female students (OR = 0.72, 95% CI = 0.122–1.623), those who spend between 2,000 and 3,000 CNY per month (OR = 0.16, 95% CI = 0.059–0.427), and those with monthly expenses of 3,000 CNY or more (OR = 0.37, 95% CI = 0.156–0.859) are less likely to engage in high-risk sexual behavior. Conversely, homosexual (OR = 3.12, 95% CI = 1.607–6.052) and bisexual (OR = 2.64, 95% CI = 1.289–5.423) individuals are more likely to engage in such behavior. Additionally, the likelihood of engaging in high-risk sexual behavior increases with both grade level and the number of romantic relationships.

**Table 4:** Binary logistic regression analysis of factors influencing high-risk sexual behavior among college students.

Variables	Category	*β*	SE	Waldχ^2^	*P*	OR (95%CI)
Gender	Male (reference)					
	Female	0.726	0.203	12.791	<0.001	0.72(0.122–1.623)
Grade	Freshman (reference)					
	Sophomore	0.687	0.296	5.386	0.02	1.99(1.113–3.548)
	Junior	0.596	0.296	4.055	0.044	1.82(1.016–3.243)
	Senior and more	0.808	0.306	6.969	0.008	2.24(1.231–4.085)
Nationality	Han (reference)					
	Minority	0.718	0.38	3.57	0.059	1.49(1.231–2.027)
Monthly expenses	≤ 1,000 CNY (reference)					
	1,000–2,000 CNY	−0.116	0.382	0.093	0.761	0.89(0.421–1.883)
	2,000–3,000 CNY	−1.841	0.506	13.267	<0.001	0.16(0.059–0.427)
	≥3,000 CNY	−1.005	0.435	5.332	0.021	0.37(0.156–0.859)
Sexual orientation	Heterosexual (reference)					
	Homosexual	1.137	0.338	11.301	0.001	3.12(1.607–6.052)
	Bisexual	0.972	0.367	7.038	0.008	2.64(1.289–5.423)
	Uncertain	0.16	0.419	0.146	0.703	1.17(0.516–2.666)
Number of love affairs	None (reference)					
	1–2 times	4.041	0.361	5.55	<0.001	4.90(1.059–8.364)
	3–5 times	2.499	0.348	11.46	<0.001	7.18(3.151–17.103)
	More than 5 times	1.21	0.47	16.637	0.001	13.35(1.336–28.42)

### 3.4 Self-reported HIV testing

The self-reported HIV testing among students participating in this survey was 4.6% (194/4,193), with a positive rate of 0.38% (16/4,193). Among students who engaged in sexual behavior, the self-reported HIV testing was 14.4% (56/388), while it was 22.0% (37/168) for those who reported high-risk sexual behavior. The results of the univariate analysis indicated statistically significant differences in self-reported HIV testing across various demographic groups for the following variables (Table 5): gender (χ^2^ = 12.353, *p* < 0.001), grade (χ^2^ = 9.814, *p* = 0.020), only child status (χ^2^ = 6.197, *p* = 0.013), nationality (χ^2^ = 21.368, *p* < 0.001), monthly expenses (χ^2^ = 33.049, *p* < 0.001), sexual orientation (χ^2^ = 68.799, *p* < 0.001), number of romantic relationships (χ^2^ = 96.004, *p* < 0.001), and sexual experience (χ^2^ = 93.176, *p* < 0.001).

**Table 5:** Univariate analysis of the factors influencing HIV testing among college students.

Variables	N (%)	HIV testing	χ^2^	*P*
Gender			12.353	<0.001
Male	1843(44.0)	109(5.9)		
Female	2,350(56.0)	85(3.6)		
Grade			9.814	0.020
Freshman	1866(44.5)	84(4.5)		
Sophomore	1,033(24.6)	47(4.5)		
Junior	881(21.0)	32(3.6)		
Senior and more	413(9.9)	31(7.5)		
Major			0.104	0.784
Medical	2,338(55.8)	106(4.5)		
Non-medical	1855(44.2)	88(4.7)		
Only child or not			6.197	0.013
Yes	1905(45.4)	105(5.5)		
No	2,288(54.6)	89(3.9)		
Nationality			21.368	<0.001
Han	4,062(96.9)	177(4.4)		
Minority	131(3.1)	17(13.0)		
Native place			0.253	0.628
Urban	2,611(62.3)	124(4.7)		
Rural	1,582(37.7)	70(4.4)		
Monthly expenses			33.049	<0.001
≤1,000 CNY	194(4.6)	18(9.3)		
1,000–2,000 CNY	2,949(70.3)	113(3.8)		
2,000–3,000 CNY	902(21.5)	45(5.2)		
≥3,000 CNY	148(3.5)	18(12.2)		
Sexual orientation			68.799	<0.001
Heterosexual	3,432(81.9)	140(4.1)		
Homosexual	85(2.0)	19(22.4)		
Bisexual	248(5.9)	19(7.7)		
Uncertain	428(10.2)	16(3.7)		
Number of love affairs			96.004	<0.001
None	3,108(74.1)	111(3.6)		
1–2 times	954(22.8)	56(5.9)		
3–5 times	51(1.2)	7(13.7)		
More than 5 times	80(1.9)	20(25)		
Sex education			0.191	0.662
Yes	3,001(71.6)	274(9.1)		
No	1,192(28.4)	114(9.6)		
Sex experience			93.176	<0.001
Yes	388(9.3)	56(14.4)		
No	3,805(90.7)	138(3.6)		

A multivariate regression analysis of self-reported HIV testing revealed that several factors significantly influenced the likelihood of HIV testing among college students. These factors included gender, whether the individual was an only child, nationality, sexual orientation, the number of romantic relationships, and sexual experience (Table 6). The analysis indicated that students who have siblings [odds ratio (OR) = 1.38, 95% confidence interval (CI) = 1.014–1.869] and those who had engaged in 1–2 relationships (OR = 2.62, 95% CI = 1.23–5.559) were more likely to undergo HIV testing. Conversely, women (OR = 0.51, 95% CI = 0.097–1.065), minority students (OR = 0.47, 95% CI = 0.258–0.864), bisexual individuals (OR = 0.18, 95% CI = 0.085–0.399), and sexually inactive students (OR = 0.42, 95% CI = 0.247–0.652) exhibited a lower likelihood of being tested for HIV.

**Table 6:** Binary logistic regression analysis of factors influencing HIV testing among college students.

Variables	Category	*β*	SE	Waldχ^2^	*P*	OR (95%CI)
Gender	Male (reference)					
	Female	0.422	0.153	5.63	0.011	0.51(0.097–1.065)
Grade	Freshman (reference)					
	Sophomore	0.087	0.245	0.127	0.722	1.09(0.675–1.764)
	Junior	0.174	0.255	0.467	0.495	1.19(0.722–1.964)
	Senior and more	0.496	0.272	3.318	0.069	1.64(0.963–2.8)
Only child or not	Yes (reference)					
	No	0.32	0.156	4.206	0.04	1.37(1.014–1.869)
Nationality	Han (reference)					
	Minority	−0.751	0.308	5.93	0.015	0.47(0.258–0.864)
Monthly expenses	≤ 1,000 CNY (reference)					
	1,000–2,000 CNY	−0.171	0.392	0.19	0.663	0.84(0.391–1.819)
	2,000–3,000 CNY	0.342	0.329	1.08	0.299	1.41(0.739–2.683)
	≥3,000 CNY	0.193	0.344	0.317	0.573	1.21(0.619–2.379)
Sexual orientation	Heterosexual (reference)					
	Homosexual	−0.143	0.295	0.236	0.627	0.87(0.486–1.544)
	Bisexual	−1.694	0.395	18.35	<0.001	0.18(0.085–0.399)
	Uncertain	−0.397	0.373	1.132	0.287	0.67(0.324–1.396)
Number of love affairs	None (reference)					
	1–2 times	0.961	0.385	6.234	0.013	2.61(1.23–5.559)
	3–5 times	0.703	0.376	3.5	0.061	2.02(0.967–4.216)
	More than 5 times	0.2	0.545	0.134	0.714	1.22(0.419–3.556)
Sex experience	Yes (reference)					
	No	−0.86	0.221	15.184	<0.001	0.42(0.247–0.652)

## 4 Discussion

This research offers a systematic and exploratory analysis of the characteristics of university students in Xuzhou City. It centers on aspects such as gender psychology, behavioral traits, and the current situation of HIV testing. Moreover, it delves deeper into exploring the crucial factors that influence high—risk sexual behaviors and the willingness of students to undergo HIV testing. The research findings show that both male and female student groups display emotional and behavioral inclinations toward same—sex attraction. Notably, the proportion of such inclinations is higher among female students compared to male students, which is consistent with the results of previous studies (23). It is noteworthy that the homosexual community is recognized as a high-risk group for HIV infection (24). According to statistics from the China CDC, the proportion of same-sex sexual transmission among newly reported HIV/AIDS cases among young students in China from 2010 to 2019 was as high as 80.0% (3). This data underscores the necessity of enhancing health education and intervention efforts targeted at sexual minorities. The study reveals that the prevalence of sexual behavior among college students in Xuzhou City is 9.3%, with a prevalence of high-risk sexual behavior at 3.27%. These figures are significantly lower than those reported in a study conducted in Sichuan City (8). This regional disparity may be attributed to several factors: firstly, the implementation of sex education in colleges and universities in Xuzhou City appears to be more effective, positively influencing student behaviors; secondly, the inclusion of medical students in the sample may contribute to a heightened awareness of health and self-protection (25). Moreover, differences in the criteria used to define high—risk sexual behavior across various studies could be a contributing factor to the disparities observed in the data.

When it comes to the factors that influence high—risk sexual behavior, the study pinpoints gender as a significant variable. It was found that women are 28.4% less likely to engage in high—risk sexual behavior compared to men (OR = 0.72, 95% CI = 0.122–1.623). This gender—based disparity can be attributed to women’s more conservative attitudes toward sexuality within the framework of traditional culture, as well as their generally more cautious approach when it comes to sexual behavior (26). These findings suggest the need for targeted sexual health education that particularly addresses the male population and enhances their awareness of sexual safety (27).

The study also indicates that the likelihood of students engaging in high-risk sexual behaviors increases with higher academic grades, with students in their fourth year or above being 1.143 times more likely (OR = 2.24, 95% CI = 1.231–4.085) to engage in such behaviors compared to freshmen. This trend may be linked to the physiological maturation and evolving sexual needs of students as they progress in their studies (28). Furthermore, the academic and employment pressures faced by senior students may also influence their behavioral choices (29). It was discovered that students with monthly living expenses surpassing 2,000 CNY are less likely to engage in high—risk sexual behaviors when compared to those with monthly living expenses below 1,000 CNY. This finding may imply a correlation between economic stress and high—risk behaviors, suggesting that students with a lower economic status might be more inclined to make high—risk sexual decisions under specific circumstances (30). The study also found that homosexual and bisexual students were 3.118 times (OR = 3.12, 95% CI = 1.607–6.052) and 2.644 times (OR = 2.64, 95% CI = 1.289–5.423) more likely to engage in high-risk sexual behaviors than their heterosexual counterparts, respectively. This finding is consistent with prior research and highlights the increased health risks faced by sexual minorities, which may be linked to their marginalized social status and vulnerability to intimate partner violence, including forced and unprotected sexual encounters (31, 32).

Finally, the findings indicate that HIV testing uptake among students who have engaged in sexual behavior increased to 14.4%, with a further rise to 22.0% among those exhibiting high-risk sexual behavior. This incremental increase in testing rates was positively correlated with students’ self-perceived risk in relation to their behavioral patterns (33). The analysis revealed that females were 0.505 times (95% CI = 0.097–1.065) more likely than males to participate in HIV testing. This gender disparity may be attributed to the social biases and psychological barriers that females often encounter in accessing medical and health screenings (34). Conversely, students who are not only children demonstrated a greater willingness to undergo HIV testing (OR = 1.38, 95% CI = 1.014–1.869). This suggests that students who have siblings may develop a heightened sense of health awareness and responsibility through familial communication and encouragement during their upbringing (35). Future research should consider integrating family dynamics into intervention strategies. Additionally, minority students exhibited a lower likelihood of undergoing HIV testing (OR = 0.47, 95% CI = 0.258–0.864), which may be attributed to cultural differences, limited access to health information, and the influence of traditional attitudes (16). This underscores the necessity of incorporating cultural sensitivity into HIV prevention initiatives and developing culturally appropriate health education materials and intervention programs tailored for ethnic minority students. Students with one to two romantic experiences were more likely to be tested for HIV (OR = 2.62, 95% CI = 1.23–5.559), suggesting that moderate emotional experiences may enhance an individual’s health awareness and self-protective behaviors (18). However, the willingness to undergo testing was notably low among bisexual students (OR = 0.18, 95% CI = 0.085–0.399), potentially due to social discrimination, identity pressures, and barriers to healthcare access faced by this demographic (36, 37). Furthermore, students with no sexual experience were less likely to be tested for HIV (OR = 0.42, 95% CI = 0.247–0.652), a trend that may reflect both plausible behavioral patterns and biased perceptions regarding HIV testing. HIV testing serves not only as a reaction to behaviors associated with elevated risk but also constitutes a vital preventive health measure and is essential for the early detection of HIV/AIDS (38). Consequently, there is a pressing need to enhance HIV prevention education for all students and to dispel the misconception that only individuals in high-risk groups require testing (39).

The above—mentioned findings offer a more exploratory and clear understanding of the current situation that university students are confronted with in terms of gender psychology, high—risk behaviors, and the challenges in the realm of HIV prevention and control. Going forward, it is absolutely crucial to intensify efforts in HIV prevention and control outreach activities. This involves the creation of a variety of health education initiatives, the provision of easily accessible testing services, and the establishment of a multi-faceted psychological support system. These measures are indispensable for effectively heightening students’ awareness of protective practices, alleviating the risk of infection, and nurturing a supportive campus environment.

## 5 Conclusion

The study indicates that, while the prevalence of high-risk sexual behaviors among students is comparatively low in relation to other regions, the HIV testing uptake remains suboptimal. Notable disparities exist in both high-risk behaviors and the willingness to undergo HIV testing among various student demographics. It is of utmost importance to direct our attention toward high—risk groups, such as upperclassmen, economically disadvantaged students, and sexual minorities. Institutions of higher learning should strengthen health education programs and provide more accessible HIV testing services. By doing so, they can ease students’ concerns and cultivate a healthy and secure campus environment.

## 6 Limitations

This study acknowledges several limitations. Firstly, it employed a cross-sectional research design, which captures data at a single point in time and is insufficient for establishing causal relationships between variables. A longitudinal study design could have better revealed the impact of time-related factors on the research outcomes. Secondly, the sample was predominantly drawn from four universities in Xuzhou City, potentially limiting the representativeness and generalizability of the findings. Thirdly, the operational definition of high-risk sexual behavior utilized in this study may differ from those in other research, which could hinder the comparability of results across different contexts. Fourth, Although this study provides some preliminary insights into HIV, it primarily relies on quantitative data and lacks exploratory qualitative analysis, failing to comprehensively explore some potential and complex social factors. Fifth, this study did not systematically analyze this complex relationship, which may underestimate the role of mental health in the health behaviors of college students. Lastly, while the study primarily examined the influence of demographic and behavioral characteristics on high-risk sexual behavior and HIV testing, it did not delve into deeper factors such as cultural background and social support. Future research should aim to enhance study design, broaden research perspectives, and focus on the dimensions of college students’ mental health to gain a more multi-faceted understanding of the challenges associated with HIV infection prevention among college students, thereby providing a scientific foundation for the development of more effective intervention strategies.

## Data Availability

The raw data supporting the conclusions of this article will be made available by the authors, without undue reservation.

## Footnotes

^1^ https://www.wjx.cn
